# Natural Modulators of Aquaporins in Cancer Therapy: Functional Mechanisms and Clinical Potential

**DOI:** 10.3390/molecules31071072

**Published:** 2026-03-25

**Authors:** Paulina Małkowska, Maciej Tarnowski

**Affiliations:** Department of Physiology in Health Sciences, Faculty of Health Sciences, Pomeranian Medical University, 71-210 Szczecin, Poland; paulina.malkowska@pum.edu.pl

**Keywords:** aquaporins, oncology, cancer, resveratrol, curcumin, bacopaside II, quercetin, EGCG, atRA, chrysin, natural compounds

## Abstract

Aquaporins (AQPs) are increasingly recognized as key regulators of tumor progression, influencing key hallmarks of cancer progression and cellular homeostasis. Their frequent overexpression in malignancies highlights their potential as therapeutic targets, yet the development of selective synthetic inhibitors remains challenging due to structural conservation and off-target toxicity. Natural compounds have recently emerged as promising modulators of AQP expression and function, offering greater molecular diversity and generally favorable safety profiles. This review synthesizes current evidence on phytochemicals, including bacopaside II, curcumin, resveratrol, quercetin, EGCG, all-trans retinoic acid, chrysin, and rottlerin, that interact with AQP isoforms relevant to cancer biology. These agents regulate AQPs through transcriptional control, redox modulation, signaling-pathway interference, or direct pore blockade, thereby attenuating oncogenic processes such as migration, angiogenesis, inflammation, and metabolic adaptation. Several compounds, notably bacopaside II and rottlerin, display isoform-selective inhibitory properties that directly impair AQP1- and AQP3-mediated permeability. Collectively, available evidence positions natural AQP modulators as promising lead compounds providing scaffolds for further drug development in oncology. Continued structural, mechanistic, and preclinical research is required to optimize isoform specificity and therapeutic efficacy, paving the way for their integration into future anticancer strategies.

## 1. Introduction

**Figure 1:**
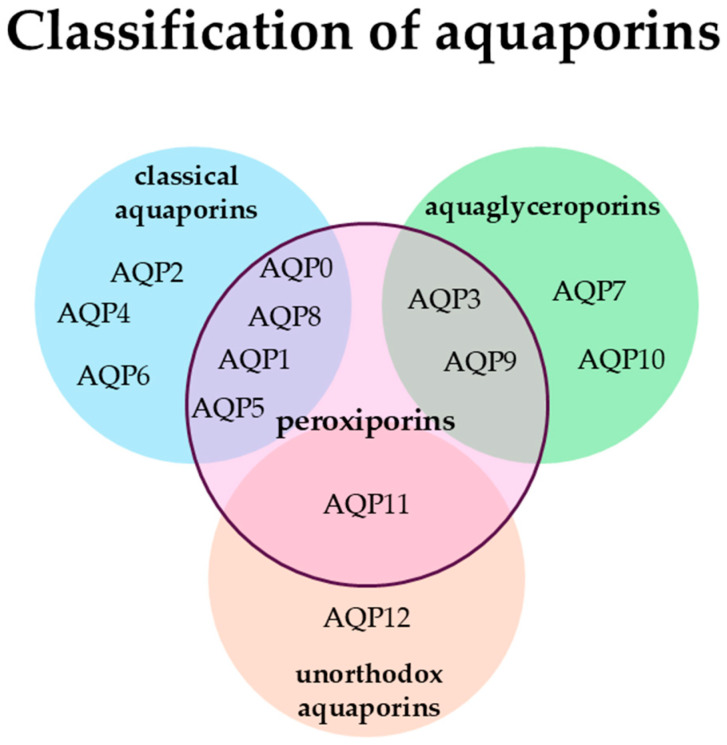
Classification scheme for aquaporins based on their primary sequence characteristics and substrate selectivity.

Aquaporins (AQPs) are a family of integral membrane proteins responsible for the transport of water and small solutes, including glycerol and hydrogen peroxide, across cellular membranes [1]. Aquaporins are functionally categorized into three main subfamilies: classical AQPs (primarily water-selective), aquaglyceroporins (permeable to water, glycerol, and small solutes), and unorthodox AQPs (with distinct subcellular localization). In the context of oncology, a particularly relevant functional subgroup is the peroxiporins (e.g., AQP1, 3, 5, 8, 11). These channels facilitate the transmembrane transport of hydrogen peroxide (H_2_O_2_), thereby acting as “transceptors” that link membrane permeability with intracellular redox signaling, a process central to tumor cell proliferation and survival (Figure 1) [2,3]. By focusing on these specific functional traits rather than broad taxonomic traits, we can better understand how natural modulators interfere with cancer-specific AQP functions.

Although originally described as key regulators of water homeostasis, AQPs have gained increasing attention in oncology due to their involvement in fundamental processes associated with fundamental oncogenic processes [4,5]. Several specific AQP isoforms, discussed below, are frequently overexpressed in malignancies and associated with aggressive tumor behavior, poor prognosis, and resistance to therapy [6,7]. Through their role in water flux, glycerol transport, and the mediation of hydrogen peroxide diffusion as peroxiporins, AQPs regulate tumor–stroma interactions, metabolic adaptation, and intracellular signaling pathways relevant to cancer progression [3].

In parallel with advances in molecular oncology, natural products have emerged as a promising source of bioactive compounds capable of modulating cancer-relevant signaling networks. Phytochemicals, flavonoids, polyphenols, alkaloids, terpenoids, and saponins have long been recognized for their anti-inflammatory, antioxidant, and antitumor effects, and many of them interact with pathways central to cell survival, EMT, migration, and angiogenesis [8,9,10]. Importantly, accumulating evidence indicates that selected natural compounds directly or indirectly regulate the expression or function of AQPs, thereby linking dietary or plant-derived molecules with AQP-dependent mechanisms of tumor progression. Examples include bacopaside II, a triterpene saponin that inhibits AQP1-mediated migration and angiogenesis [11]; curcumin, which downregulates AQP3 [12] and interferes with redox-sensitive signaling [13,14]; and quercetin, which modulates AQP4 and AQP5 in inflammation-associated models [15,16]. These findings highlight a previously underappreciated interface between natural compounds and membrane channel regulation.

Given the growing recognition of AQPs as modulators of cancer aggressiveness and the parallel emergence of natural AQP-regulating compounds as potential lead structures for drug discovery, there is a compelling need to integrate these two research areas. Understanding how natural products influence AQP isoforms and how this modulation affects oncogenic pathways may open new avenues for targeted cancer therapy and adjuvant strategies. This review summarizes the current knowledge on AQP involvement in tumor development while placing particular emphasis on natural compounds capable of modulating AQP expression or activity. Furthermore, we discuss the mechanistic foundations and translational potential of AQP-targeted interventions derived from natural sources, highlighting their promise as innovative, low-toxicity modulators of cancer progression.

## 2. Aquaporins in Cancer Development

AQPs contribute to several hallmarks of cancer, including proliferation, migration, invasion, angiogenesis, metabolic adaptation, and epithelial–mesenchymal transition (EMT) [2,17,18]. Although their canonical role involves facilitation of transmembrane water flux, selected isoforms also transport glycerol and hydrogen peroxide (H_2_O_2_), making them important regulators of cellular redox balance and metabolic homeostasis-processes frequently dysregulated in malignant tissues [3,19].

**Figure 2:**
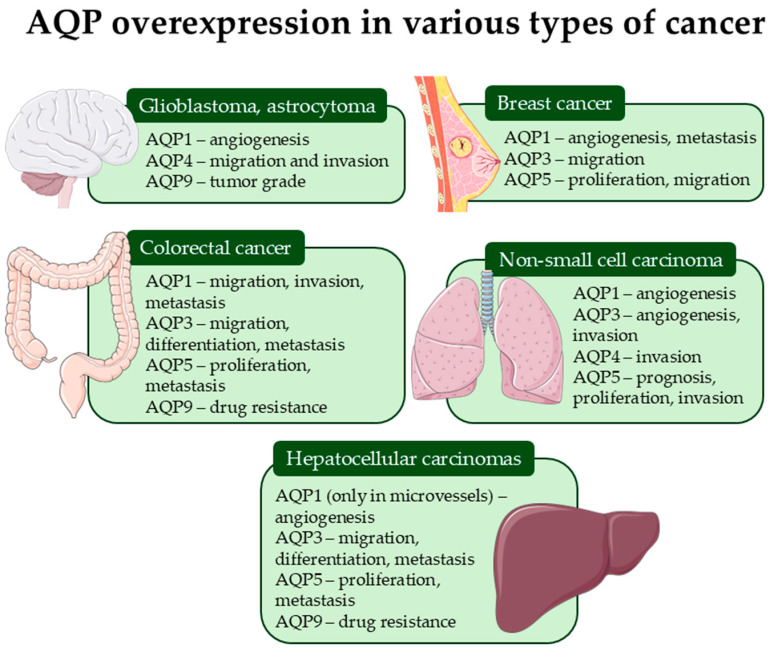
Schematic representation of aquaporin overexpression strongly associated with cancer pathophysiology.

Among the thirteen mammalian AQPs identified to date (AQP0–AQP12), isoforms AQP1, 3, 4, 5, and 9 are most extensively implicated in tumor pathophysiology across various cancer types (Figure 2). These isoforms are frequently overexpressed in malignant tissues and often correlate with poor prognosis, aggressive tumor behavior, and resistance to therapy [2]. For instance, AQP1 enhances the migratory capacity of endothelial and tumor cells through facilitation of lamellipodia formation [18], while AQP 1, 3, 4, 5, and 9 influence intracellular signaling cascades involved in proliferation and epithelial-mesenchymal transition (EMT) [20,21].

The oncogenic potential of AQPs is not solely attributed to their permeability functions, but also to their ability to modulate cellular redox status, interact with growth factor signaling pathways, and shape the tumor microenvironment. Furthermore, their role in promoting tumor angiogenesis—particularly via AQP1-mediated endothelial function—positions them as critical facilitators of tumor vascularization and growth under hypoxic conditions [22].

### 2.1. Contribution of AQPs to Angiogenesis

Angiogenesis is a critical process enabling tumor expansion beyond the diffusion limit of oxygen and nutrients. Although vascular endothelial growth factor (VEGF) and basic fibroblast growth factor (bFGF) remain the principal drivers of neovascularisation, emerging evidence indicates that several AQPS contribute significantly to endothelial migration, sprouting, and vascular remodeling [23,24].

Among these, AQP1, AQP3, AQP5, and AQP9 are the most relevant in the oncological context. AQP1, expressed in vascular endothelial cells, promotes motility and tube formation by facilitating water flux at the leading edge of migrating cells. Its deletion in murine models results in impaired angiogenesis and reduced tumor vascularization [25,26]. Additionally, as a peroxiporin, AQP1 enables the controlled transport of hydrogen peroxide (H_2_O_2_), a redox mediator that modulates hypoxia-induced angiogenic signaling and VEGF expression [22,27]. AQP3, permeable to both glycerol and H_2_O_2_ [28], supports endothelial and tumor cell migration through activation of PI3K/Akt and FGF2–ERK pathways [29], and regulates matrix metalloproteinases (MMP-2 and MMP-9), which facilitate extracellular matrix remodeling during vessel sprouting [30]. AQP5 enhances angiogenesis indirectly by amplifying proliferative and MAPK/ERK signaling in tumor cells [31,32,33], which in turn increases secretion of VEGF and other pro-angiogenic mediators [34]. AQP9 is frequently expressed in highly vascularized tumor regions [35] and participates in PI3K/Akt- and Wnt/β-catenin–dependent regulation of VEGF and matrix metalloproteinases, thereby modulating endothelial proliferation and remodeling [36].

### 2.2. Contribution of AQPs to Epithelial–Mesenchymal Transition and Loss of Cell Adhesion

Epithelial–mesenchymal transition (EMT) represents a fundamental mechanism enabling carcinoma cells to acquire motile, invasive, and therapy-resistant phenotypes [37]. While EMT is driven predominantly by hypoxia, TGF-β, inflammatory cytokines, and transcription factors such as SNAI1/2, ZEB1/2, and TWIST, accumulating evidence indicates that AQPs modulate several aspects of this process [18,38,39].

AQP1, AQP3, AQP5, and AQP9 are the isoforms most consistently linked to EMT-related phenotypes in cancer. AQP1 promotes the formation of lamellipodia and filopodia by facilitating water flux at the leading edge of migrating cells, contributing to the loss of epithelial integrity and enhanced motility [40]. Its upregulation correlates with reduced E-cadherin and increased vimentin in lung adenocarcinoma models [20]. AQP3, an aquaglyceroporin and peroxiporin, plays a central role in EMT through its ability to transport glycerol and H_2_O_2_, which activate PI3K/Akt, and redox-sensitive transcription factors [41]. AQP3 overexpression is associated with downregulation of E-cadherin and induction of mesenchymal markers in breast [42], and colorectal cancers, whereas AQP3 silencing reverses these phenotypes [43]. AQP5 drives EMT via activation of ERK1/2 and TGF-β/SMAD2 signaling and is linked to decreased epithelial markers [31] and increased vimentin levels in non-small cell lung cancer and colorectal cancer [44]. In contrast, AQP9 appears to mediate EMT suppression in hepatocellular carcinoma. Its overexpression increases E-cadherin, reduces vimentin, and inhibits proliferative and metastatic behaviors, suggesting a potential tumor-suppressive role depending on cell type [45,46].

### 2.3. Contribution of AQPs to Cancer Cell Invasion and Migration

Cell migration is a fundamental biological process essential for both unicellular and multicellular organisms, enabling tissue morphogenesis, immune defense, and wound repair [47,48,49]. The process of cellular translocation typically proceeds through several interdependent stages: polarization, protrusion, adhesion to the extracellular matrix (ECM), ECM degradation, and finally retraction of the trailing edge. Each stage is tightly controlled by cytoskeletal rearrangements, cell volume regulation, ion transport, and proteolytic remodeling of the ECM. Increasing evidence indicates that AQPs, traditionally recognized as water channels, play critical roles in modulating these events. [46,50,51,52,53].

AQP1, AQP3, AQP4, AQP5, and AQP9 are the most prominent isoforms associated with invasive behavior. AQP1 enhances motility by driving osmotic water influx at the leading edge of migrating cells, thereby supporting lamellipodial extension [50] and actin polymerization [54,55]. Loss of AQP1 disrupts cytoskeletal symmetry, reduces RhoA and Rac activity, and impairs directional migration in melanoma and breast cancer models [55,56]. AQP3 has emerged as a key mediator of hydrogen peroxide (H_2_O_2_) flux, thereby linking oxidative signaling with migratory responses. Although H_2_O_2_ is widely regarded as a reactive oxygen species, it also functions as a secondary messenger regulating proliferation, differentiation, and motility [57,58]. In cancer models, AQP3 knockdown reduced epidermal growth factor (EGF)-induced H_2_O_2_ entry, attenuated downstream signaling, and significantly impaired cell migration and growth [59]. AQP4, especially in gliomas, contributes to invasion by coordinating water efflux with Cl^−^ and K^+^ fluxes within invadopodia, facilitating ECM penetration [60,61]. Its interactions with α-syntrophin and cytoskeletal proteins further modulate actin polymerization and cell shape dynamics [62]. AQP5 increases metastatic potential through ERK1/2-dependent signaling [63,64] and cooperation with ion exchangers that aid cell movement through confined extracellular spaces [65]. AQP9 promotes filopodia formation and ECM degradation via CDC42-mediated actin remodeling and upregulation of MMP-9, particularly in prostate and colorectal cancer models [66,67].

## 3. AQPs as a Therapeutic Target for Natural Compounds

Cancer remains one of the leading causes of morbidity and mortality worldwide, and despite advances in conventional therapies such as surgery, chemotherapy, radiotherapy, and immunotherapy, treatment outcomes are often limited by resistance, relapse, and adverse effects [68]. Consequently, there is a pressing need to identify novel molecular targets that can complement existing therapeutic strategies and improve clinical efficacy. One promising avenue of research focuses on proteins that regulate the cellular processes fundamental to tumor progression, including oncogenic hallmarks.

From a mechanistic perspective, inhibition of AQPs can attenuate fundamental processes required for tumor progression. For instance, blocking AQP1 impairs lamellipodia formation and cell motility [69,70], while targeting AQP4 modulates glioma cell invasiveness and peritumoral edema [71]. Similarly, suppression of AQP3 and AQP5 reduces epithelial–mesenchymal transition (EMT), proliferation, and metastatic dissemination [43,72,73]. By interfering with these molecular events, AQP inhibition has the potential to synergize with conventional anticancer therapies, reduce metastatic spread, and improve treatment outcomes.

Despite this therapeutic promise, progress in developing clinically viable AQP inhibitors has been limited. The challenges primarily stem from the highly conserved pore architecture of AQPs, which complicates the design of isoform-selective small molecules [74]. Some inhibitors, such as tetraethylammonium, acetazolamide, or mercury chloride, show limited specificity or toxicity that preclude clinical translation [75]. More recently, innovative modalities such as monoclonal antibodies targeting extracellular loops of AQP4 (e.g., aquaporumab) in neuromyelitis optica have shown promising preclinical efficacy [76]. Nevertheless, the development of safe, selective, and effective AQP modulators remains an unmet need.

Natural products represent a compelling therapeutic research focus, as accumulating evidence indicates that their direct or indirect modulation of aquaporin function may yield beneficial anticancer effects.

### 3.1. Mechanisms of AQP Modulation by Natural Compounds

Natural products influence AQPs through several mechanistic pathways that operate at transcriptional, post-translational and functional levels. The most widely described mechanism involves the regulation of gene and protein expression (Table 1) [77]. Many phytochemicals alter transcriptional or post-translational pathways that up- or down-regulate specific AQPs. Flavonoid-rich extracts frequently modify AQP3 levels in skin, affecting hydration, wound healing and anti-aging outcomes. Ginsenoside Rg1 and curcumin suppress AQP4 over-expression, whereas honokiol up-regulates it, illustrating isoform-selective transcriptional control. Broad surveys of the literature classified the largest group of natural modulators as expression regulators, reflecting the high sensitivity of AQP genes to dietary and phytochemical signaling inputs [78].

Another proposed mechanism involves potential channel blockade. Certain plant-derived molecules bind conserved regions of the aquaporin pore and rapidly inhibit water or glycerol flux. Triterpene saponins such as bacopaside I and II have been shown to selectively block AQP1-mediated water permeability at low micromolar concentrations, with bacopaside I also inhibiting ionic conductance [79]. Additional compounds such as rottlerin, rottlerin-derived analogues and 18β-glycyrrhetinic acid derivatives directly inhibit AQP3 or AQP1, producing immediate functional effects [80]. Several flavonoids, including quercetin, which has been associated with modulation of AQP3 and related peroxiporins, and resveratrol, implicated in decreases in AQP3 expression and function via SIRT1/ERK-dependent pathways, have also been reported to affect aquaporin activity through direct or indirect inhibitory mechanisms [81,82]. However, it should be noted that in the absence of high-resolution structural evidence, these functional effects could also stem from non-specific interactions with the plasma membrane, such as changes in lipid bilayer fluidity. A smaller but mechanistically relevant group of natural compounds acts by disrupting pathological aquaporin–protein interactions. Compounds such as geraldol, tanshinone IIA and ACT001 have been described to interfere with the binding of pathogenic antibodies to AQP4, mitigating complement activation and downstream neuroinflammatory responses [83]. Although this mechanism is primarily documented in autoimmune contexts, it highlights the potential for natural agents to modulate pathological AQP interactions, which may have implications in tumor-related inflammation and edema.

### 3.2. Natural Compounds Modulating Aquaporins in Cancer

**Figure 3:**
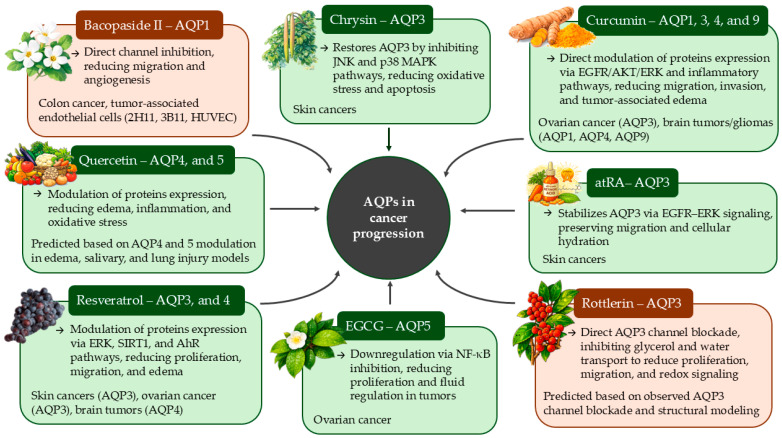
Schematic representation of the effect of natural compounds on aquaporins in different types of cancer, emphasizing the distinction between expression-based regulation (color green, e.g., EGCG, Curcumin) and functional inhibition (color orange, e.g., Bacopaside II, Rottlerin).

A variety of plant-derived compounds have been identified as modulators of aquaporin expression and function, acting through the diverse regulatory modalities summarized in Table 1 (Figure 3). The following sections highlight the most extensively characterized natural modulators of AQPs relevant to cancer biology, with emphasis on their molecular mechanisms, isoform specificity, and potential therapeutic significance.

#### 3.2.1. Bacopaside II

Bacopasides are triterpene saponins isolated from the medicinal plant *Bacopa monnieri* [84], which exhibits various pharmacological properties, including anti-cancer effects [85,86,87]. A purified extract from *Bacopa monnieri*, bacopaside II, has been identified as a natural inhibitor of AQPs. Functional studies using the *Xenopus laevis* oocyte expression system demonstrated that bacopaside II selectively impaired AQP1-mediated water transport, a process modeled as a direct interaction with the AQP1 pore, without affecting unrelated channels [11]. Its potential as an anti-cancer agent is of particular interest due to the previously described role of AQP1 in tumor angiogenesis, cell migration, and metastasis. Importantly, this inhibitory effect translated into a significant reduction in the migratory capacity of colon cancer cells, which are known to express relatively high levels of AQP1 [79]. Further insights into the pharmacological potential of bacopaside II have been obtained from endothelial cell models. Researchers investigated its effects on cell viability, apoptosis, morphology, migration, and angiogenesis-related processes, including tube formation, in mouse endothelial cell lines (2H11 and 3B11) and the human umbilical vein endothelial cell line (HUVEC). Bacopaside II demonstrated a dose-dependent inhibitory profile across these models. At a concentration of 15 μM, both murine endothelial cell lines exhibited increased apoptosis, impaired migration, and pronounced inhibition of tube formation. In contrast, at 10 μM, bacopaside II suppressed migration in both 2H11 and 3B11 cells without inducing apoptosis or reducing viability; interestingly, tubulogenesis was inhibited only in 3B11 cells, while 2H11 cells required pre-treatment at the same concentration to achieve similar inhibition [11].

HUVECs displayed the highest sensitivity to bacopaside II. At concentrations of 10–15 μM, treatment led to reduced viability, increased apoptosis, diminished migration, and suppressed tube formation. Notably, migration was significantly reduced even at 7.5 μM, without compromising cell viability, and extended pre-treatment (20 h) at this concentration further attenuated tube formation. Collectively, these findings suggest that bacopaside II not only impairs AQP1-mediated water transport but also exerts broader anti-angiogenic and anti-migratory effects on endothelial cells, reinforcing its potential utility as a therapeutic compound in cancer treatment [11].

Importantly, the compound was shown to suppress migration and tube formation in endothelial cells at sub-cytotoxic concentrations, suggesting that its inhibitory effects on angiogenesis are not solely the result of compromised cell viability. This observation is consistent with previous data demonstrating that bacopaside II significantly reduced AQP1 activity and colon cancer cell migration without affecting cell survival over 24 h [88].

#### 3.2.2. Curcumin

Curcumin, chemically described as 1,7-bis(4-hydroxy-3-methoxyphenyl)-1,6-heptadiene-3,5-dione, is a naturally occurring phenolic compound derived from the rhizomes of *Curcuma longa* and belongs to the curcuminoid family of yellow pigments [89]. For centuries, turmeric has been a cornerstone of traditional medicine, particularly in Asia, and its principal bioactive constituent, curcumin, has been widely investigated for its pharmacological properties. Extensive research has highlighted its immunomodulatory, antioxidant, anti-inflammatory, neuroprotective, and anticancer activities [90].

The anticancer potential of curcumin is particularly notable, as it has demonstrated both preventive and therapeutic benefits across a range of malignancies. Its antineoplastic effects arise from the ability to modulate key cellular and molecular mechanisms, notably by suppressing proliferation, inducing apoptosis, inhibiting angiogenesis and metastasis, and regulating inflammatory responses [91,92]. Furthermore, curcumin has been reported to specifically target cancer stem cells, a subpopulation strongly associated with therapeutic resistance and tumor recurrence, thereby offering a unique advantage over many conventional agents [89,93]. Importantly, experimental evidence suggests that curcumin may sensitize tumor cells to both chemotherapy and radiotherapy, enhancing therapeutic efficacy while mitigating adverse effects when applied as an adjuvant [90].

Despite these promising activities, the clinical utility of curcumin is constrained by its poor gastrointestinal absorption, rapid metabolism, and low systemic bioavailability [94]. To address these limitations, various strategies have been developed, including adjuvant compounds, liposomal delivery systems, curcumin nanoparticles, and phospholipid complexes. In addition, structural analogues such as EF-24 exhibit enhanced stability and improved absorption profiles, with demonstrated efficacy against cancers as well as neurodegenerative and inflammatory diseases [84]. Clinical studies further underscore its therapeutic potential; for instance, a bioavailable formulation of curcumin combined with fennel essential oil was shown to significantly improve symptoms in patients with irritable bowel syndrome [95,96].

Collectively, the pleiotropic effects of curcumin on multiple signaling cascades, including the PI3K/Akt pathway, underscore its promise as a versatile anticancer agent [90]. While substantial progress has been made in understanding its biological activities, further research is required to fully elucidate its mechanisms of action and to optimize its clinical application. Importantly, recent evidence suggests that some of curcumin’s anticancer and anti-angiogenic effects may be mediated through its influence on AQPs. This emerging line of research highlights AQPs as novel molecular targets through which curcumin may exert part of its therapeutic potential, warranting closer examination in the context of cancer biology.

Accumulating evidence indicates that curcumin modulates the expression and function of several aquaporin isoforms. In human ovarian carcinoma cells (CaOV3), AQP3 is induced by epidermal growth factor (EGF) and is required for EGF-driven cell motility; experimental knockdown of AQP3 markedly attenuates migration in this model. Crucially, curcumin downregulates basal AQP3 expression in CaOV3 cells and prevents the EGF-induced up-regulation of AQP3, thereby inhibiting EGF-stimulated migration. Mechanistically, these effects are mediated via blockade of EGFR signalling and suppression of downstream AKT and ERK phosphorylation, linking curcumin’s anti-migratory action to interruption of an EGFR → AQP3 axis [12].

Although much work on curcumin and AQPs focuses on neural injury models, the observations have clear oncological relevance where AQPs regulate tumour cell behaviour or tumour-associated oedema. For example, AQP1, highly expressed in the choroid plexus and influential in cerebrospinal fluid dynamics, has been implicated in intracranial pressure regulation after brain injury, with AQP1 deficiency improving survival and lowering intracranial pressure in animal studies. Curcumin reduces AQP1 levels in choroidal epithelial cells in a dose-dependent manner [97,98], suggesting a potential for curcumin to modulate fluid fluxes in intracranial tumours or reduce tumour-related hydrostatic stress, although direct tumour studies addressing this hypothesis are still required [97,98].

The ability of curcumin to suppress AQP4 and AQP9, as observed in various neural injury models, suggests its potential to modulate tumor-associated edema and the fluid microenvironment in neuro-oncological settings [99,100].

However, a major criticism of curcumin’s proposed mechanism is its lack of specificity and poor systemic bioavailability. While it downregulates AQP3 via EGFR/ERK inhibition, curcumin is a known “PAINS” (pan-assay interference signal) compound, meaning its observed effects may result from non-specific interactions with multiple proteins rather than a targeted AQP response [94]. Furthermore, most evidence stems from high-dose in vitro studies, which may not accurately reflect achievable concentrations in tumor tissues in vivo.

#### 3.2.3. Resveratrol

Resveratrol (3,4′,5-trihydroxy-trans-stilbene) is a naturally derived polyphenolic compound present in a range of plant sources, most notably in grapes, blueberries, and mulberries [101]. It also occurs in traditional Chinese and Japanese medicinal extracts, such as those derived from *Polygonum cuspidatum*, and has been historically used to treat inflammation, headaches, cancers, and amenorrhea [102]. Functionally, resveratrol exhibits pleiotropic effects, including anti-proliferative, antioxidant, anti-inflammatory, anti-angiogenic, and anti-metastatic activities across various cell lines [103].

At the molecular level, resveratrol acts as a modulator of multiple signaling pathways. It can inhibit aryl hydrocarbon receptor (AhR) signaling, which is linked to inflammation, apoptosis, oxidative stress, and immunosuppression—processes associated with cancer and metabolic disorders [104,105]. Additionally, resveratrol affects kinase activity, including inhibition of tyrosine kinases, and modulates MEK-ERK1/2, MAPK, NF-κB, and AP-1 pathways [106,107,108]. The compound has also been shown to engage SIRT1 signaling, contributing to its diverse biological effects, including regulation of cell proliferation and apoptosis [109,110].

Interestingly, recent studies suggest that resveratrol may influence the expression and function of AQPs, particularly AQP3, which is implicated in tumor cell migration and proliferation. AQP3 has been reported to interact with key pathways modulated by resveratrol, including ERK and AhR/ARNT signaling. These interactions position resveratrol as a potential modulator of AQP-mediated water transport and cell motility, further supporting its anti-cancer properties.

In the context of cancer, AQP3 is functionally linked to tumor cell proliferation and migration, and its expression is regulated via the ERK pathway. Resveratrol reduces ERK phosphorylation in NHEKs and other cell types, thereby downregulating AQP3 and inhibiting cell proliferation [111,112]. Notably, the inhibitory effect of resveratrol on ERK phosphorylation appears to be cell type-dependent, as studies in ovarian cancer cells suggested differential effects on upstream EGFR activation [113]. Taken together, these observations demonstrate that resveratrol can modulate AQP expression and activity in a tissue-specific manner, impacting both cancer cell behavior and neuroprotection.

Overall, resveratrol represents a versatile natural compound that targets AQPs, particularly AQP3 and AQP4, as part of its therapeutic actions. By modulating these channels, resveratrol can inhibit tumor cell proliferation and migration while simultaneously reducing brain edema and oxidative stress, highlighting the translational potential of AQP-targeting strategies in diverse pathological contexts.

#### 3.2.4. Quercetin

Quercetin is a naturally occurring bioflavonoid widely present in the human diet, found in a variety of fruits, vegetables, and beverages, including onions, broccoli, berries, apples, grapes, tea, and red wine [114,115]. It is also available as a purified compound in dietary supplements, with daily intakes reported up to 1000 mg, which may exert measurable biological effects [116]. Quercetin exhibits pleiotropic actions, including antioxidant, anti-inflammatory, anti-cancer, and anti-aging effects, as well as estrogen-like activity [117].

Preclinical studies have demonstrated that quercetin can effectively modulate AQP expression in various pathological models. Specifically, quercetin has been shown to downregulate AQP4 expression, leading to a significant attenuation of edema and reduction in pro-inflammatory cytokines such as TNF-α and IL-1β [15]. Furthermore, its ability to enhance AQP5 expression in injury models suggests a protective role in maintaining epithelial integrity and reducing oxidative damage [16,118]. Given these properties, quercetin represents a promising candidate for targeted interventions aimed at modulating AQP-mediated fluid transport and inflammatory signaling during cancer progression.

Collectively, these findings indicate that quercetin’s therapeutic effects may involve the regulation of specific AQPs, providing a mechanistic link between its anti-inflammatory, antioxidant, and anti-edematous actions. This positions quercetin as a promising candidate for interventions targeting AQP-mediated water transport in various pathological conditions, including neurovascular and epithelial tissue disorders.

#### 3.2.5. Epigallocatechin Gallate (EGCG)

Epigallocatechin gallate (EGCG), the principal catechin of *Camellia sinensis*, exhibits potent anticancer activities through the inhibition of cell proliferation, suppression of angiogenesis, induction of apoptosis, and attenuation of oxidative stress [119]. EGCG has demonstrated the capacity to suppress cell proliferation across a wide spectrum of malignancies, including cancers of the skin, lung, oral cavity, stomach, intestine, colon, liver (hepatocellular carcinoma), pancreas, rectum, prostate and breast. This broad inhibitory profile highlights its potential value as a promising anticancer agent [120].

Emerging evidence indicates that the anticancer activity of EGCG is closely linked to its capacity to modulate aquaporin expression through NF-κB–dependent regulatory pathways. In ovarian cancer models, EGCG downregulates AQP5 in a concentration- and time-dependent manner, and this reduction correlates strongly with impaired cellular proliferation [121,122,123]. Mechanistically, EGCG enhances IκBα stability and prevents its degradation, thereby inhibiting the nuclear translocation of the NF-κB p65 subunit, a pathway previously implicated in the regulation of tumorigenesis, invasion, and metastasis in ovarian cancer [124,125]. The resulting suppression of NF-κB transcriptional activity diminishes the expression of NF-κB–regulated genes, including those controlling the long-term transcriptional regulation of AQP5, whose promoter contains NF-κB-, AP-1-, and AP-2–responsive elements [120]. This mechanistic relationship is further supported by studies showing that pharmacological inhibition of NF-κB using PDTC reduces both AQP5 mRNA and protein expression while simultaneously suppressing SKOV3 cell proliferation. Consistent with these findings, EGCG-induced decreases in AQP5 expression are positively correlated with reduced nuclear levels of p65 and altered IκB expression (r = 0.968–0.995; *p* < 0.05), indicating that the modulation of AQP5 by EGCG occurs through an NF-κB–mediated mechanism [120]. Collectively, these data demonstrate that EGCG targets AQP5 by disrupting NF-κB–driven transcriptional programs, thereby inhibiting AQP-dependent fluid regulation associated with tumor growth and ascites formation and contributing to apoptosis in ovarian cancer cells.

Despite the strong correlation between EGCG-mediated NF-κB inhibition and AQP5 downregulation, the precise direct target remains elusive. It is debated whether EGCG acts primarily on AQP5 transcription or whether the reduction in AQP5 expression is a secondary consequence of the compound’s broader antioxidant effects and overall reduction in cellular stress [120].

#### 3.2.6. All-Trans Retinoic Acid (atRA) and Chrysin

All-trans retinoic acid (atRA), the biologically active metabolite of vitamin A [126], and chrysin (5,7-dihydroxyflavone), a naturally occurring flavonoid found in propolis, honey, and various plant sources, have attracted growing interest due to their combined antioxidant, anti-inflammatory, and anti-tumor properties [127]. Although both compounds are widely studied in dermatological and photoaging contexts, their mechanistic actions on aquaporin-mediated signaling pathways provide a compelling rationale for evaluating their therapeutic relevance in oncology.

Both atRA and chrysin have demonstrated the capacity to restore the expression and function of aquaporin-3 (AQP3) under stress conditions, a process with significant implications for tumor cell motility and microenvironmental interactions [127,128,129]. atRA exerts its regulatory effect through a dual mechanism involving the trans-activation of the epidermal growth factor receptor (EGFR) and the concurrent inhibition of MEK/ERK signaling. By stabilizing AQP3 levels, atRA preserves cellular hydration and limits aberrant ERK activation, thereby potentially restraining the migratory capacity and aggressive phenotype of certain cancer cells [128].

Chrysin similarly modulates AQP3 expression, primarily through the suppression of stress-activated MAPKs, including JNK and p38. Since these signaling pathways play central roles in cancer cell survival, metastatic potential, and inflammatory responses, chrysin-mediated AQP3 stabilization may indirectly contribute to anticancer effects by restraining pro-tumorigenic stress responses [127]. Collectively, the mechanistic link between atRA, chrysin, and AQP3 regulation provides a biologically plausible framework for their evaluation as adjunctive strategies in AQP-centered cancer therapy [129].

#### 3.2.7. Rottlerin

Rottlerin (RoT) is a naturally occurring polyphenolic compound isolated from the mature fruits of *Mallotus philippinensis*, a plant widely used in traditional medicine across several Asian regions. Extracts of this species are rich in bioactive phenolic constituents that exhibit antimicrobial, antioxidant, antiviral, immunomodulatory, and anticancer activities [80]. Among these natural compounds, RoT has garnered particular attention due to its potent antitumor properties demonstrated across a broad spectrum of human cancers [130]. RoT is recognized as a multifunctional molecule capable of exerting antiproliferative, antiangiogenic, anti-inflammatory, anti-allergic, antimicrobial, antifungal, antiparasitic, and ROS-quenching activities [80]. Importantly, unlike many polyphenols with broad cytotoxic profiles, RoT displays minimal toxicity in non-tumorigenic cells both in vitro and in vivo [131,132,133,134], thereby supporting its suitability as a therapeutic candidate.

Recent findings provide the first evidence that RoT acts as a functional inhibitor of aquaporin-3 (AQP3), an aquaglyceroporin implicated in tumor growth, redox regulation, and metastatic progression [135]. Given the increasing interest in AQPs as modulators of cancer biology and therapeutic targets, the identification of RoT as an AQP3 inhibitor places this compound within a growing class of phytochemicals capable of regulating AQP expression or activity with potential clinical benefit [81,136]. Functional assays demonstrate that RoT inhibits AQP3-mediated water and glycerol permeability in human erythrocytes, with micromolar-range IC_50_ values. Notably, its stronger inhibition of glycerol flux compared to water permeation aligns with the higher IC_50_ measured for water transport and reflects the differential gating and pore architecture of AQP3. Additional experiments in yeast cells expressing human AQP1 or AQP3 confirm RoT’s specificity: while AQP3 activity is markedly reduced, AQP1 remains unaffected, underscoring the selectivity of the compound [80].

Based on computational models, it has been suggested that RoT might act as a channel blocker. Computational modeling combined with molecular docking reveals that RoT interacts with several key residues located on the extracellular surface of the AQP3 pore. The compound’s benzenotriol and chromenol moieties form stable contacts that effectively cap the pore entrance, while the flexible phenylpropyl chain engages hydrophobic residues near the aromatic selectivity filter. This constellation of interactions produces a stereochemical “lid” that obstructs glycerol access to the pore without entirely preventing water passage. The structural specificity and stability of this binding configuration support a high-affinity inhibitory mechanism that does not rely on upstream modulation of protein trafficking or kinase activity, despite RoT’s established role as a kinase inhibitor [80]. While molecular docking predicts a ‘capping’ effect on the pore, these findings require validation through mutagenesis or structural analysis to rule out indirect effects on the membrane environment.

The discovery of this direct inhibitory effect on AQP3 provides a compelling explanation for RoT’s broad anticancer profile. By blocking AQP3-mediated glycerol and, to a lesser extent, water transport, RoT disrupts metabolic, osmotic, and redox pathways that support tumor proliferation, migration, and survival. Because AQP3 overexpression is characteristic of many aggressive tumors, particularly those exhibiting enhanced glycerol metabolism or redox-driven signaling, RoT’s selective AQP3 blockade positions it as a promising lead compound for AQP-targeted cancer therapy, serving as a molecular template for the synthesis of more potent and selective derivatives. Further refinement of the molecule, guided by the structural determinants identified in binding simulations, may yield RoT derivatives with even greater specificity and potency, advancing the development of clinically applicable AQP3 inhibitors.

While computational modeling suggests rottlerin acts as a direct “pore-blocker” for AQP3, this mechanism is criticized due to the compound’s well-known role as a potent protein kinase C (PKC) inhibitor. Distinguishing between direct channel blockade and indirect effects caused by the inhibition of AQP phosphorylation and subsequent trafficking remains a significant challenge for validating rottlerin as a selective AQP3-targeted lead [80].

## 4. Conclusions

AQPs have emerged as critical regulators of oncogenic processes, including proliferation, migration, invasion, angiogenesis, redox signaling and epithelial–mesenchymal transition. Their frequent overexpression in malignant tissues, together with their functional contributions to tumor aggressiveness, underscores their relevance as therapeutic targets. However, the development of selective and clinically applicable AQP inhibitors has been significantly constrained by structural conservation within the AQP family and by the limited specificity and safety of currently available synthetic modulators.

In this context, natural compounds provide a promising reservoir of bioactive molecules capable of modulating AQP expression or function through diverse mechanisms. Evidence summarized in this review demonstrates that phytochemicals exert regulatory effects on AQP isoforms implicated in cancer progression through two primary modalities: (1) modulation of gene and protein expression (e.g., EGCG, curcumin, resveratrol) and (2) putative functional inhibition of channel permeability (e.g., bacopaside II, rottlerin). These effects encompass transcriptional modulation, suppression of oncogenic pathways, redox regulation, and, in some cases, putative pore blockade, as suggested by functional and docking studies for bacopaside II (AQP1) and rottlerin (AQP3). Importantly, many of these agents achieve functional inhibition of cancer cell motility, proliferation, angiogenesis, or inflammation without inducing substantial toxicity in non-malignant cells, highlighting their potential translational value.

Collectively, the evidence supports the view that natural AQP modulators constitute a diverse pool of lead compounds with the potential to inform the design of future AQP-targeted drugs. A critical gap persists between promising in vitro observations and clinical applicability. Future research must address current criticisms by prioritizing structural studies and mutagenesis to confirm direct ligand-AQP interactions and utilizing more sophisticated in vivo models. Overcoming the challenges of isoform specificity and bioavailability is essential to transition these natural modulators from experimental tools into validated, safe components of precision cancer therapy. While these phytochemicals offer a unique starting point, they currently serve as experimental tools and lead structures rather than clinical-grade therapeutics. Future efforts must focus on synthetic optimization to overcome their inherent limitations in bioavailability and isoform specificity.

## Figures and Tables

**Table 1:** Summary of natural AQP modulators and their primary mechanisms of action.

Compound	Target AQP(s)	Primary Mechanism	Effect on AQP
**Bacopaside II**	AQP1	Functional (Putative pore blockade)	↓ Water permeability
**Rottlerin**	AQP3	Functional (Putative pore blockade)	↓ Glycerol/Water flux
**Curcumin**	AQP1, 3, 4, 9	Expression (Transcriptional/Signaling)	↓ Protein/mRNA levels
**Resveratrol**	AQP3, 4	Expression (SIRT1/ERK signaling)	↓ Protein expression
**Quercetin**	AQP4, 5	Expression (Redox/Inflammatory signaling)	↓/↑ Tissue-specific levels
**EGCG**	AQP5	Expression (NF-κB inhibition)	↓ mRNA/Protein expression
**atRA/Chrysin**	AQP3	Expression (EGFR-ERK/MAPK stabilization)	↑ Recovery of expression

↓—decrease; ↑—increase.

## Data Availability

Data sharing is not applicable.
